# Blood Cell Ratio Biomarkers of Non-type 2 Inflammation in Chronic Obstructive Pulmonary

**DOI:** 10.21203/rs.3.rs-8585567/v1

**Published:** 2026-01-20

**Authors:** Kaman So, Aabida Saferali, Jeong Yun, Min Hyung Ryu, Enrico Schiavi, Peter Castaldi, Lisa Ruvuna, Russell Bowler, Jeffrey Curtis, Craig Hersh

**Affiliations:** Brigham and Women's Hospital; Brigham and Women's Hospital; Brigham and Women's Hospital; University of British Columbia; Catholic University of the Sacred Heart; Brigham and Women's Hospital; Cleveland Clinic; Cleveland Clinic; University of Michigan–Ann Arbor; Brigham and Women's Hospital

**Keywords:** COPD exacerbation, Lymphocytes, Neutrophils, Platelets, Proteomics, RNA-sequencing

## Abstract

**Background:**

The majority of COPD patients are characterized by non-type 2 inflammation, yet there are no available non-type 2 biomarkers, as opposed to blood eosinophil count for type 2 inflammation. We aimed to test readily obtainable immune cell ratios as biomarkers for clinical phenotypes in COPD and to determine pathways represented by these ratios using multi-omics data.

**Methods:**

Using complete blood counts with differential collected at the Phase 2 (5-year) visit in the COPDGene Study, we calculated three immune cell ratios previously described in COPD and other diseases: the neutrophil-lymphocyte ratio (NLR), the platelet-lymphocyte ratio (PLR), and the Systemic Immune-Inflammation Index (SII = NLR*platelets). We tested for associations with COPD outcomes, including lung function, chest CT scan phenotypes, and exacerbations. Blood RNA-sequencing and proteomics data were used to identify genes, proteins and pathways associated with the ratios.

**Results:**

In univariate analyses, the three biomarkers were associated with COPD severity measures. In zero inflated Poisson regression models, all three were associated with increased odds of having an exacerbation but were not associated with exacerbation counts. Conversely, the three biomarkers were generally associated with prospective exacerbation counts, but not the zero-inflation term. In logistic regression models, the three biomarkers were significantly associated with having two or more exacerbations in the prior year; however, receiver operating characteristic analyses did not lead to clear cutoff values. Complement and PI3K signaling pathways were enriched across more than one ratio in both the RNA-sequencing and proteomics results. Other inflammatory pathways relevant in COPD appeared in different enrichment sets in either omics data type.

**Conclusions:**

Higher levels of three easily obtained blood cell ratios were associated with COPD severity and exacerbations outcomes; however, there are not clear thresholds which would be required for clinical application. Blood RNA-sequencing and proteomics identified inflammatory pathways associated with the three biomarkers, including targets for COPD therapies currently in human trials.

## Background

Chronic obstructive pulmonary disease is a heterogeneous condition, yet there are limited biomarkers available to define the different inflammatory pathways.(1) Blood eosinophil count, a biomarker for type 2 inflammation, is associated with exacerbation risk(2) and response to inhaled corticosteroids,(3) and has been used as an entry criteria into clinical trials of biologic therapies targeting type 2 inflammation in COPD.(4, 5) Fractional exhaled nitric oxide and immunoglobulin E levels are other biomarkers of type 2 inflammation that are used in clinical care for asthma and have been applied in research studies of COPD.(5, 6) Despite the clinical relevance of type 2 inflammation in COPD, this mechanism is likely important only in a minority of COPD patients. Studies have shown that 20–40% of COPD patients have evidence of type 2 inflammation, indicated by elevated blood eosinophil counts.(2, 7) Non-type 2 inflammation is defined only by the absence of elevated blood eosinophils; there are no available biomarkers for this relevant COPD mechanism.

Complete blood count with white blood cell differential is a commonly obtained clinical laboratory measurement. Both blood neutrophil counts and platelet counts are elevated in inflammatory states and are associated with COPD severity.(8, 9) Several blood cell ratios have been developed to capture systemic inflammation, including the neutrophil to lymphocyte ratio (NLR), the platelet to lymphocyte ratio (PLR) and the systemic inflammatory-immune index (SII = NLR * platelets). These ratios have been predominantly applied in cancer studies, but are increasingly shown to be relevant for COPD outcomes including exacerbations and mortality.(10-13) These indices are purported to reflect systemic inflammation, but the specific inflammatory pathways represented are unknown.

Our hypothesis is that these blood cell ratios can serve as biomarkers of non-type 2 inflammation. We executed two aims to test this hypothesis. First, we sought to confirm the associations with respiratory outcomes, especially exacerbations, in a large population of current and former smokers with and without COPD in the Genetic Epidemiology of COPD Study (COPDGene). Second, we aimed to identify the biological pathways represented by the indices using blood RNA-sequencing and proteomics data in COPDGene.

## Methods

### The Genetic Epidemiology of COPD Study (COPDGene)

COPDGene is an observational study of over 10,000 current and former smokers (at least 10 pack years) with and without COPD, along with additional non-smoking controls.(14) One-third of COPDGene subjects are African Americans. Subjects were ages 45–80 at the initial visit. Subjects with cancer within the last 5 years and with other lung diseases except asthma were excluded. The baseline study visit included questionnaires, spirometry, a 6-minute walk test, and a high-resolution chest computed tomography (CT) scan with computerized analysis for emphysema and airway disease. Five- and ten-year follow-up visits (Phases 2 and 3) used similar phenotype assessments, with the addition of a complete blood count (CBC) with differential, which was assayed in each center’s clinical laboratory. COPD exacerbations in the past year are queried at each study visit, and prospective exacerbations are recorded using web or telephone-based longitudinal follow-up questionnaires every 6 months.(15) COPD exacerbations were defined by use of antibiotics and/or systemic steroids. Severe exacerbations required emergency department visits or hospitalization. Frequent exacerbators were defined as two or more exacerbations in the prior year. All subjects gave written informed consent. COPDGene was approved by the Institutional Review Boards at all participating centers.

### RNA-sequencing and proteomics

At the Phase 2 visit, blood samples were collected for RNA-sequencing and proteomics; methods have been previously published.(16, 17) Briefly, whole blood was collected in PaxGene RNA tubes (BD Biosciences). RNA was extracted and used to generate stranded total RNA libraries with ribosomal reduction. Illumina sequencing used 75bp paired-end reads. Blood proteomics was generated using SomaScan v4.0 which uses aptamers to quantify approximately 5000 proteins. Whole genome sequencing is available through the NHLBI Trans-Omics in Precision Medicine (TOPMed) program.(18) Duffy null phenotype was determined by minor allele homozygosity for a single nucleotide polymorphism in the atypical chemokine receptor 1 gene (*ACKR1*; rs2814778).(19)

## Statistical analysis

All statistical analyses were performed in R (v.4.3.2) in R Studio Pro Server (v2025.05.1 + 513). Unless otherwise stated, to test the univariate associations of NLR, PLR, and SII with clinical variables, we used the 2-tailed unpaired Student’s t test for categorical variables, and correlation for quantitative variables. To test the association between three immune cell ratios and exacerbations counts, zero-inflated Poisson regression analyses were performed using the pscl (v.1.5.9) package in R. Zero-inflated Poisson models were adjusted for age, sex, race, current smoking status, pack-years, FEV_1_ percent predicted and Duffy null phenotype, the major determinant of neutrophil count primarily in populations of African descent.(20) Scale function was applied to the predictors, which generally improves model stability and convergence, especially for predictors with large values or different scales. To test the association between the three immune cell ratios and binary exacerbation outcomes, logistic regression analyses were performed using stats (v.4.2.0) package in R. Logistic models adjusted for age, sex, race, smoking status, pack-years, FEV_1_ percent predicted and Duffy null phenotype. Models for prospective exacerbations were additionally adjusted for the number of exacerbations in the prior year.

We performed differential gene expression and proteomic analysis using Limma (v3.54.2) in R. Linear models were adjusted for age, sex, race, smoking status, pack-years, and FEV_1_ percent predicted. As per previous COPDGene analyses, proteomics were additionally adjusted for clinical center,(17) and RNA-seq was also adjusted for library batch.(16) We used Sigora (v3.1.1) to identify the underlying function of gene sets. Four annotated gene sets -- hallmark gene sets,(21) KEGG,(22) Gene Ontology(23) and Reactome(24) v2025.1 -- were chosen for the reference gene sets. An FDR adjusted p-value < 0.05 was set as the cutoff criteria.

## Results

Neutrophil-lymphocyte ratio (NLR), platelet-lymphocyte ratio (PLR), and systemic immune-inflammation index (SII) were derived from Phase 2 CBC data for 5,652 subjects, including 1,970 with COPD, defined as post-bronchodilator FEV_1_/FVC < 0.7 and FEV_1_ < 80% predicted, corresponding to GOLD stages 2–4 (Table 1). The three measures were highly correlated: NLR-PLR (Pearson) r = 0.66, NLR-SII r = 0.89, SII-PLR r = 0.76, in all subjects.

In univariate analyses, all three biomarkers were significantly associated with demographic and clinical variables such as sex, race, smoking status, age, lung function (FEV_1_% predicted), disease-related quality of life (St. George’s Respiratory Questionnaire [SGRQ] total score), exercise capacity (6-minute walk distance), and chest CT scan emphysema measures in all subjects and in COPD only (Table 2); overall, the correlations were weak. NLR and SII, but not PLR, were associated with chest CT scan airway metrics, in all subjects and in COPD only. Weak negative correlations were found between blood eosinophil counts and NLR (r = −0.03) and PLR (r = −0.07); SII was not significantly correlated (r = −0.009). Moderate repeated measures correlations were observed between Phase 2 and Phase 3 values for NLR (r = 0.34, n = 2844), PLR (r = 0.53, n = 2826), and SII (r = 0.35, n = 2826).

Exacerbations were assessed both retrospectively and prospectively. Subjects were followed for an average of 7.05 (± 2.65) years. In zero inflated Poisson regression models for exacerbations in the past year, adjusted for clinical covariates and Duffy null genotype, higher values for all three biomarkers were associated with reduced odds for being a non-exacerbator (zero inflation term in Table 3A) – i.e. higher odds of having an exacerbation -- but were not associated with exacerbation counts. Conversely, the three biomarkers were generally associated with prospective exacerbation counts, but not the zero inflation term. Similar results were found for prospective severe exacerbation counts (data not shown). In logistic regression models adjusted for covariates, the three biomarkers were significantly associated with having frequent exacerbations, defined as two or more in the prior year (Table 3B). All three were associated with having a severe exacerbation in the past year; however only NLR and SII were associated with having a severe exacerbation in the prospective follow-up.

**Figure 1:**
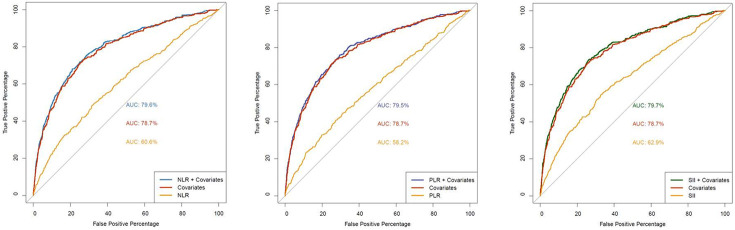
Receiver operating characteristic curves for frequent exacerbations outcome (2 or more in the prior year)

To assess the discrimination of frequent exacerbation status (two or more in the prior year), we next constructed receiver operating characteristic (ROC) analyses. When combined with clinical covariates, all three biomarkers showed small but significantly increased area under the ROC curve (AUROC) values compared to covariates alone in all subjects (Fig. 1, Table 4). In COPD subjects, AUROCs were slightly lower than in all subjects (Supplemental Fig. 1, Table 4); NLR and SII showed significant improvement compared to covariates alone. We examined various cutoffs to dichotomize the three biomarkers, using the 1st, 2nd or 3rd quartile of each. ROC curve analyses did not show an optimal cutoff in all subjects or in COPD subjects only (Supplemental Table 1)

### Blood RNA-sequencing and Proteomics

Using whole blood RNA-sequencing data we found that the majority of the expressed transcripts were significantly associated (false discovery rate < 0.05) with each of the three blood cell ratios: NLR 13,458 transcripts, PLR 13,380, SII 13,307 (Supplemental Tables 2–4). A large number of plasma proteins were also significantly associated with each ratio, although these represented a lower fraction of the total proteins assayed: NLR, 1429 proteins; PLR, 1602; SII, 1807 (Supplemental Tables 5–7). Pathway enrichment analysis of the top 300 genes in each analysis (ranked by adjusted p-value) was performed using several databases including Gene Ontology, KEGG, Reactome, and Hallmark gene sets (Table 5, Supplemental Tables 8–13). As positive controls, we employed pathways related to neutrophil degranulation (NLR and SII RNA-seq and proteomics) and platelet activation (PLR RNA-seq and proteomics). In the RNA-seq analysis, there was enrichment across more than one cell ratio in several pathways, including apoptosis, Rho GTPase, complement, and IL6/JAK/STAT3 signaling. Similarly, in the proteomics data, there was enrichment across more than one ratio in pathways related to collagen and extracellular matrix, epithelial-mesenchymal transition, interleukin signaling, complement, and PI3K signaling. Thus, the latter two pathways appeared in both RNA-seq and proteomics analyses. Other inflammatory pathways relevant in COPD appeared in different enrichment sets, including signaling pathways such as TNF-alpha, WNT, and IL-2/STAT5. Interestingly, IL-4/IL-13 signaling, relevant for T2 inflammation, was enriched in the SII RNA-seq and NLR proteomics results. A number of targets for COPD therapies currently in human trials appeared in the enriched proteomics pathways, such as neutrophil elastase, alpha-1 antitrypsin, and IL1RL1 (ST2 protein).(25, 26)

## Discussion

We examined three blood cell ratios – neutrophil-lymphocyte ratio, platelet-lymphocyte ratio, and systemic immune-inflammatory index -- in a large study of current and former smokers with and without spirometry-defined COPD. We found that these ratios were orthogonal to blood eosinophil count, a currently used biomarker in COPD management. They were associated with almost all COPD severity outcomes, including retrospective and prospective exacerbations, though it is not clear whether they add substantially to clinical risk factors for exacerbations. Using RNA-sequencing and proteomics obtained at the same blood draw, we showed that these ratios were associated with multiple biologic pathways, including inflammatory pathways relevant for COPD.

The three ratios, especially the NLR, have been extensively studied in multiple chronic diseases in addition to COPD. Previous studies have demonstrated associations with COPD outcomes, including acute exacerbations. Studies of NLR and PLR have been reviewed elsewhere.(10, 27, 28) Despite the multiple studies, no clear consensus has emerged on an optimal cut-off which would be required to use a ratio as a clinical biomarker. Our study largely confirmed that higher levels of these biomarkers are associated with exacerbations and similarly did not find an optimal threshold value for any of the three ratios. We controlled for multiple clinical covariates including lung function, and found a statistically significant, but unlikely clinically relevant, improvement in discriminative ability in the ROC curve analyses.

The SPIROMICS study, another large observational study of COPD in the US, has recently reported associations between NLR and outcomes in current and former smokers with and without COPD, as well as non-smokers.(29) They found stability in NLR values at 6 weeks and 1 year (intraclass correlations 0.74 and 0.62, respectively). Subjects with NLR in the highest quartile had increased odds of an exacerbation within the following year and higher mortality. They found no difference in mortality for quartiles 1–3, but did not examine these thresholds for associations with exacerbations, as we did. Similar to our study, the SPIROMICS analyses were adjusted for Duffy null phenotype.

Our study addressed three easily obtained blood cell ratios in a large sample with and without COPD, finding consistent results regardless of disease status. The novelty of our study is the assessment of two different omics data types, blood RNA-sequencing and proteomics, allowing us to determine the biological pathways that may be indicated by these biomarkers. Reassuringly, we found strong enrichment for neutrophil and platelet-related pathways. Several inflammatory pathways were found in one of the two omics datasets. Complement and phosphatidylinositol 3-kinase (PI3K) signaling were shared by more than one biomarker in each of the two omics data types.

Proteins in the complement pathway are known to be altered in COPD.(30) Proteomics analysis of blood and bronchoalveolar lavage fluid found enrichment for proteins in the complement pathway in subjects with rapid lung function decline.(31, 32) Markers of complement activation are elevated in sputum during COPD exacerbations.(33) Hypocomplementemic urticarial vasculitis, a rare small vessel vasculitis, has been associated with COPD.(34)

PI3K signaling is involved in fundamental cellular processes such as growth and proliferation. Several PI3K inhibitors are FDA-approved for use in different types of cancer.(35) PI3K activation leads to secretion of inflammatory cytokines and generation of reactive oxygen species by several immune cell types in COPD, including neutrophils.(36) PI3K is a potential target for COPD therapies.(37)

Our study has several limitations. In COPDGene, CBC with differential were obtained starting at the Phase 2 (5-year) visit, not at the baseline visit. Despite this, we still have a large sample size with an average of 7 years follow-up time. Furthermore, the RNA-seq and proteomics data were collected concurrently at Phase 2. The blood cell ratios may not fully reflect lung inflammation; bronchoalveolar lavage or sputum samples would be required for comparison and were not included in COPDGene.

## Conclusions

The Global Initiative for Chronic Obstructive Lung Disease (GOLD) includes measurement of blood eosinophil count as part of the initial assessment of a person with COPD.(1) The three ratios we analyzed can be derived from the same CBC with differential that is used to measure the blood eosinophil count. Therefore, there is the potential to gain additional information without additional testing. Currently, COPD patients are dichotomized into type 2 and non-type 2 inflammation subtypes using the blood eosinophil count. However, it is likely that both pathways, and other inflammatory pathways, are active to varying degrees in individual patients. Further studies will be needed to determine how these biomarkers can be used to determine response to existing therapies targeting type 2 inflammation – including inhaled corticosteroids and biologics – as well as novel therapies targeting other inflammatory mechanisms.

## Supplementary Material

This is a list of supplementary files associated with this preprint. Click to download.

- Supplement01.12.26.docx
- SupplementalTables1113Proteomicspathways.xlsx
- SupplementalTables27Differentialexpression.xlsx
- SupplementalTables810RNAseqpathways.xlsx

## Figures and Tables

**Table 1:** COPDGene Phase 2 subject characteristics. N(%) or mean (SD) are shown

	All Subjects(n = 5652)	COPD Only(n = 1970)
Female sex	2799 (49.5%)	898 (45.6%)
Non-Hispanic white	3930 (69.5%)	1493 (75.%8)
African American	1722 (30.5%)	477 (24.2%)
Current smoker	2194 (38.8%)	677 (34.4%)
Age, years	65.26 (8.66)	67.76 (8.24)
Pack-years of smoking	43.70 (24.09)	51.66 (25.06)
FEV1% predicted, post-bronchodilator	77.81 (24.54)	52.27 (17.22)
SGRQ total score	23.47 (21.31)	36.32 (21.03)
6 min walk distance, ft.	1290.99 (436.86)	1113.35 (434.12)
BODE score	1.68 (2.15)	3.32 (2.48)
% emphysema (LAA950)	5.42 (9.02)	11.86 (12.43)
Perc15	84.81 (30.36)	66.18 (29.25)
Pi10	2.28 (0.58)	2.69 (0.56)
Airway wall thickness of segmental airways	1.04 (0.22)	1.13 (0.23)
Wall area % of segmental airways	50.29 (8.43)	54.77 (7.98)
White blood cell count	7.18 (2.34)	7.56 (2.31)
Neutrophil count	4.33 (1.80)	4.76 (1.95)
Lymphocyte count	2.06 (1.12)	1.95 (0.87)
Monocyte count	0.56 (0.21)	0.6 (0.22)
Eosinophil count	0.19 (0.15)	0.2 (0.16)
Exacerbations in the prior year	4652 (82.3%)	1336 (67.8%)
0	581 (10.3%)	350 (17.8%)
1	419 (7.4%)	284 (14.4%)
2 or more		
Severe exacerbation in the prior year	532 (9.4%)	361 (18.3%)

Abbreviations:

BODE: Body mass index, Obstruction, Dyspnea, Exercise Capacity

FEV_1_: forced expiratory volume in 1 second

LAA950: low attenuation area at −950 Hounsfield Units

Perc15: 15th percentile of lung density histogram

Pi10: square root wall area of hypothetical airway with 10mm internal perimeter

SGRQ: St. George’s Respiratory Questionnaire

**Table 2:** Univariate associations of NLR, PLR, and SII with clinical variables Categorical variables (mean values are shown)

	All subjects			COPD		
Variable	NLR	PLR	SII	NLR	PLR	SII
Male	2.63**	132.1	594.1	3.12**	141.9	715.9
Female	2.29	134.0	590.7	2.66	142.9	967.8
White race	2.70**	136.9**	648.6**	3.12**	145.2*	763.0**
African American race	1.90	124.0	461.7	2.24	133.3	533.1
Current smoker	2.19**	121.9**	539.4**	2.49**	126.8**	614.3**
Former smoker	2.63	140.1	625.8	3.12	150.4	756.1

* p < 0.05

** p < 0.001

A. Quantitative variables (correlation coefficients are shown)

**Table 3:** Associations with exacerbation outcomes Zero inflated Poisson regression models for exacerbations counts (beta (SE) are shown)

		All subjects			COPD		
Outcome	Term	NLR	PLR	SII	NLR	PLR	SII
Exacerbations in past year	Zero inflation	−0.22 (0.05)**	−0.22 (0.05)***	−0.25 (0.05)***	−0.18 (0.07)**	−0.21 (0.07)**	−0.24 (0.09)**
	Count	0.001 (0.018)	−0.001 (0.018)	0.002 (0.016)	0.02 (0.03)	−0.004 (0.03)	0.003 (0.02)
Prospective exacerbations	Zero inflation	0.14 (0.09)	0.28 (0.08)***	0.13 (0.08)	0.32 (0.21)	0.28 (0.17)	0.38 (0.21)
	Count	0.43 (0.01)***	0.37 (0.01)***	0.39 (0.01)***	0.65 (0.01)***	0.42 (0.02)***	0.54 (0.01)***

* p < 0.05

** p < 0.01

*** p < 0.001

A. Logistic regression models for frequent exacerbations (2 or more in the prior year) and severe exacerbations (beta (SE) shown)

**Table 4:** Area under the receiver operating characteristic curve, frequent exacerbation outcome (2 or more in the prior year)

	Covariates only	NLR	NLR +covariates	PLR	PLR +covariates	SII	SII +covariates
All subjects	78.7	60.6	79.6^a,b^	58.2	79.5^a,b^	62.9	79.7^a,b^
COPD only	74.6	57.1	75.4^a,b^	57.7	75.4^b^	59.7	75.6^a,b^

a p<0.05 for comparison vs covariates only by Delong test

b p<0.0001 for comparison with biomarker only (e.g. NLR) by Delong test

Abbreviations

NLR: neutrophil to lymphocyte ratio

PLR: platelet to lymphocyte ratio

SII: systemic immune-inflammation index

**Table 5:** Selected results from pathway analysis of RNA-sequencing and proteomics data. Full results are available in Supplemental Tables 7–30.

	RNA-sequencing			Proteomics		
Pathway	NLR	PLR	SII	NLR	PLR	SII
Complement		**	**	**	**	**
PI3K signaling			**	**		**
Apoptosis	**		**			
RhoGTPase	*	**	*			
Epithelial-mesenchymal transition				**	**	**
Collagen/extracellular matrix				**	**	**
IL6/JAK/STAT3 signaling	**		**	**		
IL2/STAT5 signaling			*			**
TNF-alpha signaling			**			
WNT signaling				**		
IL4/IL13 signaling			**	**		

* Bonferroni adjusted p < 0.05

** Bonferroni adjusted p < 0.001

## Abbreviations

AUROC: area under the ROC curve
BODE: Body mass index, Obstruction, Dyspnea, Exercise Capacity
CBC: complete blood count
CT: computed tomography
FEV_1_: forced expiratory volume in 1 second
FVC: forced vital capacity
LAA950: low attenuation area at −950 Hounsfield Units
NLR: neutrophil to lymphocyte ratio
Perc15: 15th percentile of lung density histogram
Pi10: square root wall area of hypothetical airway with 10mm internal perimeter
PLR: platelet to lymphocyte ratio
ROC: receiver operating characteristic
SGRQ: St. George’s Respiratory Questionnaire
SII: systemic immune-inflammation index
